# Linkage and mapping of quantitative trait *loci* associated with angular leaf spot and powdery mildew resistance in common beans

**DOI:** 10.1590/1678-4685-GMB-2015-0314

**Published:** 2017-02-20

**Authors:** Denis Bassi, Boris Briñez, Juliana Santa Rosa, Paula Rodrigues Oblessuc, Caléo Panhoca de Almeida, Stella Maris Nucci, Larissa Chariel Domingos da Silva, Alisson Fernando Chiorato, Rosana Pereira Vianello, Luis Eduardo Aranha Camargo, Matthew Wohlgemuth Blair, Luciana Lasry Benchimol-Reis

**Affiliations:** 1 Centro de Recursos Genéticos Vegetais, Instituto Agronômico de Campinas (IAC), Campinas, SP, Brazil; 2 Centro de Grãos e Fibras, Instituto Agronômico de Campinas (IAC), Campinas, SP, Brazil; 3 Centro Nacional de Pesquisa de Arroz e Feijão, EMBRAPA, Santo Antônio de Goiás, GO, Brazil; 4 Departamento de Fitopatologia, Escola Superior de Agricultura Luiz de Queiroz (ESALQ), Universidade de São Paulo (USP), Piracicaba, SP, Brazil; 5 Department of Agriculture and Environmental Sciences, Tennessee State University, Nashville, TN, USA

**Keywords:** Pseudocercospora griseola, Erysiphe polygoni, quantitative inheritance, SSRs, SNPs

## Abstract

Angular leaf spot (ALS) and powdery mildew (PWM) are two important fungi diseases causing significant yield losses in common beans. In this study, a new genetic linkage map was constructed using single sequence repeats (SSRs) and single nucleotide polymorphisms (SNPs), in a segregating population derived from the AND 277 x SEA 5 cross, with 105 recombinant inbred lines. Phenotypic evaluations were performed in the greenhouse to identify quantitative trait *loci* (QTLs) associated with resistance by means of the composite interval mapping analysis. Four QTLs were identified for ALS resistance. The QTL ALS11^AS^, linked on the SNP BAR 5054, mapped on chromosome Pv11, showed the greatest effect (R^2^ = 26.5%) on ALS phenotypic variance. For PWM resistance, two QTLs were detected, PWM2^AS^ and PWM11^AS^, on Pv2 and Pv11, explaining 7% and 66% of the phenotypic variation, respectively. Both QTLs on Pv11 were mapped on the same genomic region, suggesting that it is a pleiotropic region. The present study resulted in the identification of new markers closely linked to ALS and PWM QTLs, which can be used for marker-assisted selection, fine mapping and positional cloning.

## Introduction

Common bean (*Phaseolus vulgaris* L.) represents an important source of protein in the human diet, especially in developing countries (Gepts *et al.*, 2008). The species is cultivated in several countries around the world, and Brazil is the second leading producer and the largest consumer (FAOStat, 2014). Angular leaf spot (ALS) caused by *Pseudocercospora griseola* (Sacc.) Crous & Braun (sin. *Phaeoisariopsis griseola* (Sacc.) Ferraris) (Crous *et al.*, 2006) severely reduces common bean yield in tropical and subtropical regions. This disease occurs in more than 60 countries including Brazil, and depending on the environmental and management conditions the losses can reach up to 80% (Schwartz *et al.*, 1982; Jesus Júnior *et al.*, 2001). This disease causes necrotic lesions in the leaves, pods, and stems. Lesions may also appear on the seeds, resulting in losses in grain productivity and quality.

Powdery mildew (PWM), caused by *Erysiphe polygoni* DC (Ferreira *et al.*, 1999) is another disease that causes serious damage to bean crops. Although it has a worldwide distribution, it is considered a secondary disease (Sartorato *et al.*, 1996). However, the incidence of this disease has increased in recent years, mainly due to increased planting of winter crops, where environmental conditions are favorable to the development of pathogens (Rezende *et al.*, 1999). Losses can reach 69%, mainly when the infection occurs before the anthesis (Hall, 1991). Initial symptoms are characterized by small round spots on the leaves or stems, which grow and form a whitish mycelial mass at later stages of infection, covering the entire plant (Schwartz *et al.*, 2005).

Among the various strategies of management, the most efficient and economical one has been the use of resistant cultivars. However, the high genetic variability observed in these pathogens has facilitated the development of different physiological races (Schwartz, 1994; Pastor-Corrales and Jará, 1995; Silva *et al.*, 2008), and consequently, it is difficult to obtain varieties with large degree of resistance. Resistant sources to angular leaf spot have been identified (Pastor-Corrales *et al.*, 1998; Mahuku *et al.*. 2003; Sartorato, 2006), of which the majority were described with monogenic dominant or recessive inheritance pattern (Carvalho *et al.*, 1998; Gonçalves-Vidigal *et al.*, 2011; Mahuku *et al.*, 2011). Cultivar AND 277 is distinguished by possessing *Phg*-1, *Phg*-2^2^, *Phg*3^2^ and *Phg*-4^2^ alleles that confer resistance to nine races of angular leaf spot, which include the races 63.23 and 63.19, frequently found in Brazilian planting areas (Alzate-Marin *et al.*, 2003; Caixeta *et al.*, 2005; Reis-Prado, 2006).

Sources of resistance to PWM have also been described (Schwartz *et al.*, 1981), including ‘Cornel 49242’, ‘Porrillo Sintético’, ‘Negro San Luis’ and ‘ESAL 686’ cultivars (Rezende *et al.*, 1999; Trabanco *et al.*, 2012; Pérez-Vega *et al.*, 2013). Much of these sources are characterized by possessing a few genes involved in the trait with different patterns of action.

In addition to the studies that observed qualitative genetic inheritance, there is also evidence of quantitative trait *loci* (QTLs) controlling ALS resistance (López *et al.*, 2003; Mahuku *et al.*, 2009, 2011; Oblessuc *et al.*, 2012, 2013, 2015; Keller *et al.*, 2015). Five QTLs were mapped on linkage group Pv04, one on Pv08, another on Pv09 and three on Pv10 (López *et al.*, 2003; Mahuku *et al.*, 2009, 2011). Mahuku *et al.* (2011) identified two resistance genes on the G10909 cultivar. In addition, Caixeta *et al.* (2005) observed by alelism tests three genes (*Phg-3, Phg-4* and *Phg-5*) with two alleles each, controlling the resistance in four cultivars (‘AND 277’, ‘Mexico 54’, ‘MAR 2'and ‘Cornell 49-242’) that were previously characterized as having only one resistance gene QTL associated to PWM resistance (Melo *et al.*, 2002; Hanai *et al.*, 2010). These results strengthen the evidence that the type of genetic inheritance involved in the resistance to ALS and PWM is more complex than that described by several authors, and additional studies need to be conducted to better understand these host–pathogen relationships.

Molecular-genetic maps and QTL mapping are tools that allow the localization of some genomic regions that control both single and complex inheritance, making possible the study of the genetic architecture of the traits of interest (Lynch and Walsh, 1998), such as resistance to diseases. From a breeding perspective, it is interesting to have maps fully saturated with markers, indicating genes and/or QTLs locations (Hanai *et al.*, 2010). This information could be used in breeding programs for producing new cultivars by marker-assisted selection and for helping breeders understand the effects and mode of action of *loci* that control the traits of interest.

Several linkage maps have been constructed for *P. vulgaris* (Blair *et al.*, 2007; Grisi *et al.*, 2007; Campos *et al.*, 2011; Oblessuc *et al.*, 2014). The construction of new maps using populations that have not been previously mapped is interesting for integrating mapping studies, synteny analysis, and discovering and validating new QTLs.

In this study, we aimed to (1) validate effective ALS and PWM resistance *loci* in common beans and (2) develop closely linked markers for breeding applications.

## Materials and Methods

### Plant material

The mapping population was composed of 105 recombinant inbred lines (RILs) in the F_8_ generation. This population was obtained by the crossing between AND 277 and SEA 5 cultivars at the International Center for Tropical Agriculture (CIAT, Cali, Colombia). SEA 5 belongs to the Mesoamerican gene pool and it is susceptible to angular leaf spot. Singh *et al.* (2001) registered the line SEA 5 as a drought tolerant cultivar, derived from interracial crosses between the races Mesoamerican and Durango, and one of the parents originating the line was the cultivar BAT 477, also described by the authors as drought tolerant. Later, Terán and Singh (2002) also observed productive superiority of the genotype SEA 5 in both water deficit and under irrigated condition, using BAT 477 and San Cristobal 83 as tolerant controls. Studying the root system by means of a screening using soil tube system to evaluate the impact of drought on different genotypes of beans, Rao *et al.* (2006) found that SEA 5 and BAT 477 remained among the genotypes with deeper roots. SEA 5 was also used in studies for drought tolerance and other traits of agronomic interest (Blair *et al.*, 2006).

AND277 from the Nueva Granada race belongs to the Andean gene pool (Blair *et al.*, 2009) and it was also developed at CIAT (Cali, Colombia). Cultivar AND 277 [Cargabello x (Pompadour ChecaxLínea 17) x (Línea 17 x Red Kloud)] is an important resistance source used in breeding programs in Brazil and Southern Africa (Carvalho *et al.*, 1998, Gonçalves-Vidigal *et al.*, 2011). AND 277 has the *Co*-*1* ^*4*^ allele that confers resistance to *C. lindemuthianum* (Arruda *et al.*, 2008) and the *Phg*-*1* ALS-resistance gene that confers resistance to some Brazilian *P. griseola* races (Caixeta *et al.*, 2005). In greenhouse evaluations, AND 277 showed resistance to races 63.23 e 63.19 known as severe and highly frequent in Brazilian bean field. (Reis-Prado, 2006). SEA 5 x AND 277 population also detains contrast in relation to drought tolerance and was used in mapping studies in common bean (Briñez Rodriguez, 2013). This same breeding population was used in evaluating drought tolerance in greenhouse conditions (data not shown).

### Characterization and genotyping of the AS population with SSRs

Genomic DNA was isolated from the RIL and parental leaves, following the protocol described by CIMMYT (2005). A total of 328 SSRs (Hanai *et al.*, 2007; Campos *et al.*, 2011) were characterized and the ones that were polymorphic in the parents were selected to construct the molecular-genetic map. PCR products were separated in polyacrylamide gel electrophoresis (PAGE) (6%) and revealed by silver staining.

### SNP genotyping

A total of 384 SNPs, previously identified for *P. vulgaris* (Müller *et al.*, 2015) polymorphic between BAT 93 (Mesoamerican) and JALO EEP558 (Andean) lines, was genotyped by Vera Code^®^ technology with Bead X press platform (Illumina) and selected to compose the oligopool assay (OPA).

Three oligonucleotides were used for each of the variations of the same SNP and the third specific-locus binding to the 3’ region of the DNA fragment containing the target SNP, generating a unique allele-specific fragment. Subsequently, this fragment was amplified using *Taq* DNA polymerase enzyme Titanium (Clontech®) and complementary primers labeled with *Cy*3 and *Cy*5 fluorophores.

Genotyping was realized by Genome Studio software version 1.8.4 (Illumina, EUA) using Call Rate values ranging from 0.80 to 0.90 and GenTrain ≥ 0.26 for SNP grouping. Automated analyses were performed to cluster the SNP alleles of each line, based on the signal intensity for *Cy*3 and *Cy*5 fluorophores, resulting in three genotype classes, AA, BB, and AB. Groups were adjusted individually and manually by determining the best clusters based on the parental profile.

### Linkage map construction

Segregation analysis for 105 RILs and parents was done by Chi-Square test and *p-*values associated with the test were calculated using R statistical software (version 2.12.2, R Development Core Team, 2011). The genetic map was constructed by OneMap software, version 2.0-1 (Margarido *et al.*, 2007), using multipoint approaches and Markov models, adopting a likelihood of odds (LOD-score) limited ratio of 3.0 and maximum genetic distance of 37.5 cM as thresholds by using the Kosambi mapping function (Kosambi, 1944).

The molecular markers’ probable physical location in the chromosomes was verified by BLASTN analysis (Altschul *et al.*, 1997) using the *P. vulgaris* genome (https://phytozome.jgi.doe.gov/pz/portal.html; Schmutz *et al.*, 2014) and by comparisons with integrated genetic maps for the common bean, based on SSRs mapping (Blair *et al.*, 2011; Campos *et al.*, 2011). The nomenclature described by Pedrosa-Harand *et al.* (2008) was used. The design of each linkage group with markers in their respective positions and distances was done with MapChart 2.2 program (Voorrips, 2002).

### Angular leaf spot and powdery mildew evaluations

The *P. griseola* isolates were obtained from naturally ALS-infected bean leaves collected from the Agronomic Institute (IAC, Campinas, SP. Brazil) fields, in different bean growing areas, and characterized into races based on their reactions in the twelve internationally differential bean cultivars (Pastor-Corrales and Jara, 1995).

Inoculation (2 × 10^4^ spores mL^-1^) was conducted during the period in which plants reached the V3 phenological stage, in an acclimatized room. Plants were kept at a relative humidity (RH) > 95% and temperature of 22 °C for 48 h and then transferred to the greenhouse. Symptoms were evaluated 15 days after inoculation. Plants were scored for disease severity using a 1–9 scoring scale (Van-Schoonhoven and Pastor-Corrales, 1991). Parental evaluation was performed in the same experiment as checks. Digital analysis using ImageJ^®^ software (Rasband, 2014) was also processed, considering the number of lesions, lesion area (cm^2^), and percentage affected.

A completely randomized greenhouse block design was used, with four replications and plots consisting of boxes of 29.5 cm x 46.5 cm x 12.5 cm, filled with commercial substrate (Plantmax^®^) prepared with pine bark. Seeds from three different RILs were planted in three rows in each box, with each one corresponding to a recombinant inbred line. Rows consisted of four plants, spaced approximately 4 cm from each other, resulting in 12 plants per box. NPK 04-14-08 fertilization was performed at a dose of 400 L ha^-1^. Carioca cultivar was also used as checks. Infection caused by *E. polygoni* occurred naturally. Disease severity evaluations occurred thirty days after planting and performed with the aid of diagrammatic notes developed by Blum *et al.* (2003), based on infection percentage (Table S1).

### Statistical analysis

The average of notes generated from four plants per RIL per block corresponded to the final disease score of each line. These values were used for analysis of variance and F tests using the general linear models (GLM) procedure, using SAS software v.8.2 (SAS Institute, Cary, NC, USA). Broad sense heritability (h^2^) was estimated according to Falconer and Mackay (1996). In order to confirm the contrasting resistance profile among genotypes, separate analyses were performed for the parents and recombinant inbred lines. Effects of different sources of variation were considered significant by F test when P ≤ 0.05. Skewness, kurtosis (Mardia, 1970) and Shapiro and Wilk (1965) normality tests were applied to verify normal distribution of variance analysis residuals.

### Mapping resistance loci associated to angular leaf spot and powdery mildew

QTL Cartographer software v1.17 (Basten *et al.*, 2005) was used, applying composite interval mapping with model selection based on Bayesian Information Criterion for choosing the best model, including or excluding the main effects of QTLs (Zeng *et al.*, 1999).

Likelihood ratio tests (LRT) were used to verify the presence and effect of identified QTLs. LOD values were calculated using the formula LOD = 0.2172 x LRT. Multiple linear regression for each linkage group position was applied considering the level of significance equal to α = 0.05.

The significance value for detection of QTLs was determined by performing tests with 1000 permutations (Churchill and Doerge, 1994). R2 values and additive effects of each QTL were identified. Positive effect values are related alleles that increase the susceptibility, while negative effects are related to the action of resistance alleles.

### Location and functional analyses of markers linked to QTLs

The genome location of all markers present in the interval of QTLs was verified by the alignment to the bean genome available on Phytozome v10.3 database (https://phytozome.jgi.doe.gov/pz/portal.html). The criteria used to assign putative regions to the markers included E-values ≤ 1×10^−10^ and minimum identity of 50% between query and database sequences. The closest transcripts to each marker were annotated for their putative function, with the goal of analyzing the genomic context of the QTLs mapped.

## Results and Discussion

### AS population genotyping

A total of 150 (46%) SSRs were polymorphic between the AS parents. A similar polymorphism ratio was observed in other studies (Yu *et al.*, 2000; Grisi *et al.*, 2007). Among the polymorphic SSRs markers, 24% were of composite-type, 20% dinucleotides, 24% trinucleotides, and 13% tetranucleotides. It is important to consider the nature of the SSR motives, such as length and number of repeat units to select the best markers suited for genotyping (Garcia *et al.*, 2011).

For SNPs, 288 (75%) were polymorphic between the AS parents. Among them, 9% were heterozygous, and were thus not included in the mapping analysis. According to the literature, there is a broad distribution of SNPs throughout the bean genome (Gaitán-Solís *et al.*, 2008; Souza *et al.*, 2012).

### Construction of the AS map

**Figure 1:**
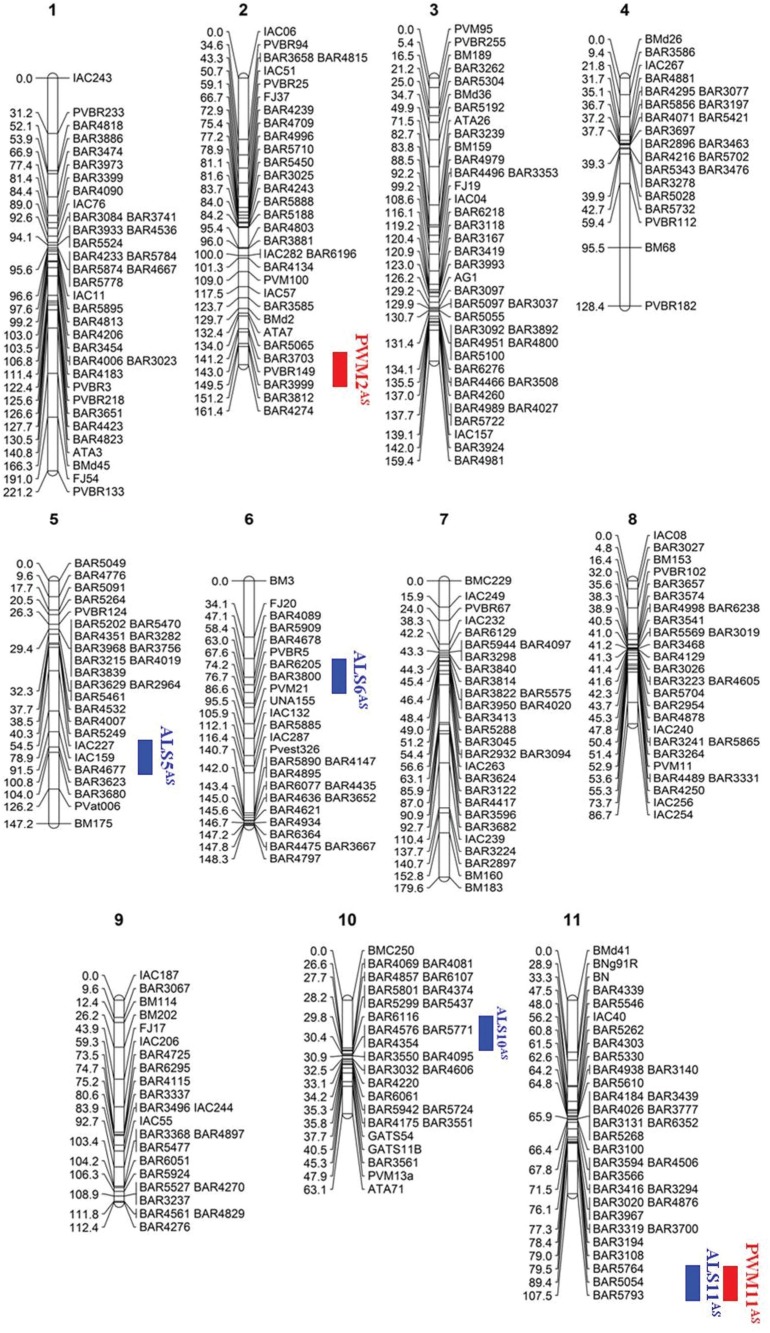
Genetic map for common bean derived from linkage analysis between 79 SSRs and 252 SNPs. Red bars indicate powdery mildew resistance QTLs while blue bars represented angular leaf spot resistance QTLs.

All the SSRs and SNPs showed the expected Mendelian segregation ratio of 1:1. Among the 150 polymorphic SSR markers, 80 (53% of total) were used in the mapping analysis. A total of 251 SNP markers were used in the analysis due to the exclusion of those with heterozygous profiles. In total, 331 markers were linked to the 11 chromosomes (Figure 1) of common beans, resulting in a map length of 1,515.2 cM and an average distance between markers of 4.5 cM.

The SNPs and SSRs were distributed in all linkage group chromosomes (Table 1), ranging from 17 (Pv09) to 31 SNPs (Pv11), and 4 (Pv11) to 11 SSRs (Pv02). The size of the bean chromosomes ranged from 63.1 cM (Pv10) to 221.2 cM (Pv01). Pv04 and Pv09 presented the lowest number of linked *loci*, while Pv03 and Pv01 showed the highest number of linked markers. The order of the markers on Pv03 was maintained when compared with previous studies (Cordoba *et al.*, 2010; Campos *et al.*, 2011; Garcia *et al.*, 2011). In addition, BLASTN analysis confirmed the correct association of markers on common bean chromosomes using the Phytozome database.

**Table 1:** Distribution of SSRs and SNPs, number of *loci*, linkage group length and average distance between markers in the genetic map developed from the AND277xSEA5(AS) population using OneMap^®^software.

Linkage group	SSR	SNP	N° of *loci*	Length (bp)	Average distance between *loci* (cM)
Pv01	10	26	36	221.2	6.1
Pv02	11	21	32	161.4	5.0
Pv03	10	30	40	159.4	3.9
Pv04	5	18	23	128.4	5.5
Pv05	5	22	27	147.2	5.4
Pv06	8	19	27	148.2	5.4
Pv07	8	22	30	179.6	5.9
Pv08	7	22	29	86.7	2.9
Pv09	7	17	24	112.4	4.6
Pv10	5	23	28	63.1	2.2
Pv11	4	31	35	107.5	3.0
Total	80	251	331	1515.2	4.5

In the AS map, it was possible to associate 276 new *loci*, with no gaps between them, providing a new tool for synteny studies, map integration, and mapping of agronomically important traits.

### Identification of physiological races of *P. griseola* and parental characterization

Each isolate collected in the Agronomic Institute fields corresponded to a different race of Mesoamerican origin (isolate IAC-1, race 1.21; isolate IAC-2: race 1.5; isolate IAC-3: race 0.22). These different races in the collecting area may be due to the high genetic variability observed within the species (Sartorato, 2002; Mahuku *et al.*, 2003).

All races caused symptoms in the susceptible SEA 5 parent. However, race 1.21, (isolate IAC-1) caused the most severe symptoms and therewith, it was chosen to be used in the disease response evaluations of the whole mapping population. The average severity score measured among the four AND 277 parental plants was 1, characterizing it as highly resistant. The average severity score of SEA 5 was 6.2, characterizing it as susceptible.

The parameters [number of lesions, lesion area (cm^2^), and leaf affected percentage (%)] evaluated with ImageJ^®^ for the AND 277 parent were all 0, showing high resistance; unlike the SEA 5 parent, where the number of lesions (27), lesion area (19.35 cm^2^), and leaf affected percentage (52.48%), revealed susceptibility to angular leaf spot. Therewith, processing and analyzing of digital images (Figure S1) confirmed the resistance and susceptibility profiles of AND 277 and SEA5, respectively.

These results were consistent with the levels of resistance reported in other studies for AND 277 (Aggarwal *et al.*, 2004; Reis-Prado, 2006; Gonçalves-Vidigal *et al.*, 2011), making it a promising line for common bean breeding programs aiming for ALS resistance.

### Disease evaluation of the AS population

Normality test (skewness, kurtosis, and Shapiro-Wilk) results were not significant, indicating normal distribution for residuals associated with analysis of phenotypic values of ALS and PWM symptoms evaluations (Skewness ALS, P = 0.66; kurtosis ALS, P = 0.94; Shapiro–Wilk ALS, P = 0.98; skewness PWM, P = 0.16; kurtosis PWM, P = 1.71; Shapiro–Wilk PWM, P = 0.98)

Analysis of variance and F test for severity of ALS and PWM detected significant differences between parents and RILs. The high variability between the lines was confirmed by highly significant values for the F test (F-value equal to 2.3 and 12.6, P < 0.0001 to ALS and PWM, respectively), for all genotypes, supporting the hypothesis that the AS population is representative for mapping resistance *loci*.

The contrasting parental profile in relation to ALS resistance was again evidenced in the assay with the 105 RILs (Table 2). The same profile was found for PWM; however, SEA 5 behaved as moderately resistant and AND 277 as susceptible (Table 2).

**Table 2:** Estimates of means, standard deviations and broad sense heritabilities for angular leaf spot and powdery mildew resistances.

Genotypes	Angular leaf spot	Powdery mildew
AND 277	1.1 ± 1.0*	5.6 ± 0.8*
SEA 5	3.4 ± 1.0*	3.8 ± 0.8*

* Significant differences at 0.05 of probability

Broad sense heritability to ALS resistance was considered moderate to high (Table 2). Similar values were also estimated in other studies. Oblessuc *et al.* (2012) estimated a value of 0.69 in greenhouse assays. Miklas *et al.* (2001) observed a value of 0.65 to white mold resistance. According to Amaro *et al.* (2007), the values of heritability estimated to ALS resistance are usually high, allowing phenotypic selection for recombination to be performed in the F_2_ generation.

The estimate of broad sense heritability for PWM was higher than ALS resistance (Table 2). Kasettranan *et al.* (2010) estimated values of 0.94 in field conditions and 0.92 in greenhouse assays for PWM heritability, using RIL populations. These values suggest that the PWM resistance trait suffers less environmental influence favoring gains with few selection cycles.

Transgressive segregation in resistance and susceptibility to both studied diseases was observed (Figure S2), which provides evidence for the presence of minor genes for the resistance to these diseases in both SEA 5 and AND 277. Oblessuc *et al.* (2012) also observed transgressive segregation to ALS in greenhouse and field assays. One possible cause for the occurrence of transgressive segregation is the presence of complementary genes with additive effects within the parents (Beebe *et al.*, 2008) that, when combined, result in higher or lower phenotypic expression.

### QTLs associated with resistance to ALS and PWM

**Figure 2:**
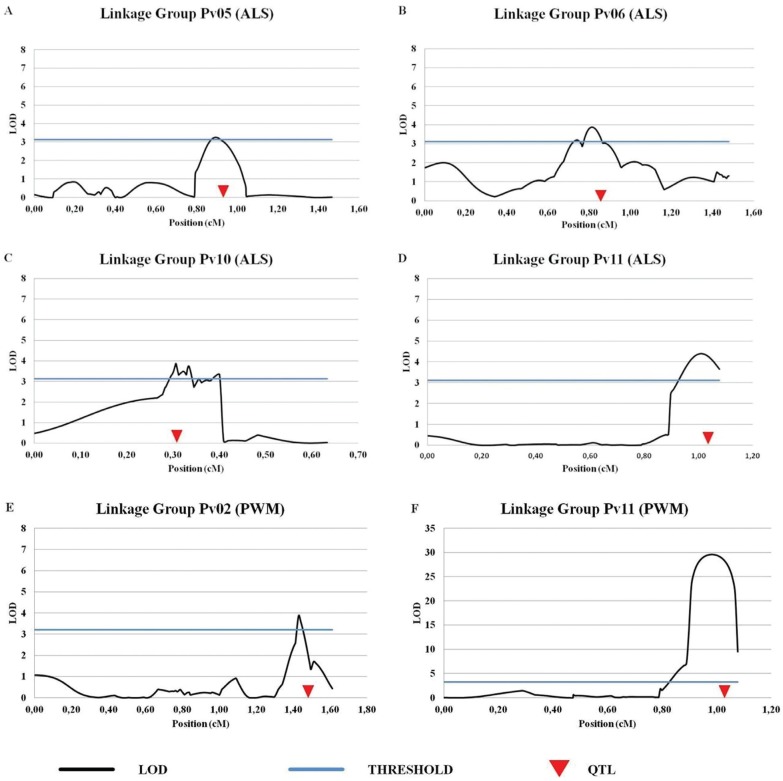
QTL likelihood plots found by CIM analysis for the identification of resistance QTLs mapped in the genetic map developed from the AND 277 x SEA 5(AS) population. QTLs associated to angular leaf spot (ALS) were referred as A to D and QTLs associated to powdery mildew (PWM) were referred as E and F.

Threshold values obtained by permutation analysis revealed six resistance QTLs for both studied diseases (Figures 1 and 2), of which four were associated with ALS and two with PWM, mapped on Pv02, Pv05, P06, Pv10, and Pv11. Three QTLs were mapped in regions covering SNP markers such as the BAR3800 which was the marker closest to the maximum LOD score for the ALS6^AS^, mapped between BAR6205 and PVM21 markers, on Pv06. For Pv10, the BAR5771, located between BAR576 and BAR4354 was the marker closest to the maximum LOD score for the QTL ALS10^AS^. The BAR5054 marker was also located on the ALS11^AS^ and PWM11^AS^ QTL peaks, on Pv11, between BAR5764 and BAR5793 markers. Two QTLs were mapped in these regions, covering SSR markers, such as the IAC159, located between IAC227 and BAR4677 markers on Pv05, closest to the maximum LOD score for the ALS5^AS^, and the PVBR149 located on Pv2, between BAR3703 and BAR3999 markers, closest to the maximum LOD score for the PWM2 ^AS^ (Table 3).

**Table 3:** QTLs to angular leaf spot and powdery mildew resistance mapped in the AND 277 x SEA5 RIL common bean population using 80 SSRs and 251 SNPs.

Disease	LG	QTL	Interval (cM)	Marker	LOD	Additive Effect	R2 (%)
ALS	Pv05	ALS5^AS^	79.2-104.3	IAC159	3.26	0.38	15.3
ALS	Pv06	ALS6 ^AS^	67.6-98.5	BAR3800	3.86	-0.36	14.4
ALS	Pv10	ALS10^AS^	21-40	BAR5771	3.87	-0.35	13.7
ALS	Pv11	ALS11^AS^	78.6-107.7	BAR5054	4.39	-2.45	26.5
PWM	Pv02	PWM2 ^AS^	136-149.5	PVBR149	3.88	-0.47	7.3
PWM	Pv11	PWM11^AS^	79.3-107.7	BAR5054	29.6	1.53	66.5

LG = Linkage group ALS = Angular leaf Spot PWM= Powdery Mildew

Several resistance QTLs to bean white mold have been identified on Pv2, Pv5, and Pv6 (Kolkman and Kelly, 2003; Ender and Kelly, 2005; Miklas, 2007). Jung (1998) mapped the gene that confers resistance to rust (*Pu*) on Pv5. Corrêa *et al.* (2001) mapped resistance genes to rust on Pv10. Navarro *et al.* (2007) identified resistance QTLs associated with bacterial brown spot on Pv6 and Pv11. Campa *et al.* (2014) identified a complex cluster of bean anthracnose resistance genes at the end of Pv11. Oblessuc *et al.* (2012) identified a major QTL associated to angular leaf spot resistance on Pv10 (ALS10.1). The putative R genes cluster at ALS10.1 was shown to be down-regulated in the infected susceptible parent (IAC-UNA) suggesting its contribution to plant susceptibility to the fungus (Oblessuc *et al.*, 2015).

In our study, a major effect QTL associated to ALS resistance was the ALS11^AS^, located on Pv11, which explained 26% of the phenotypic variance (Table 3), while the ALS5^AS^, mapped on Pv5, explained 15% of the phenotypic variation.

For PWM, the PWM11AS resistance QTL had a major effect on the resistance, explaining 66% of the phenotypic variation (Table 3). This *locus* also presented the highest LOD value (29.6), which strongly supports superior accuracy. Ferreira *et al.* (1999) reported that reactions of common bean genotypes against powdery mildew under controlled conditions provided clear evidence about the qualitative nature of resistance involving different modes of inheritance. This qualitative nature was supported in this study by the detection of a major effect QTL (PWM11AS). For PWM, other resistance genes were mapped at the end of Pv04 and Pv11 (Trabanco *et al.*, 2012; Pérez-Vega *et al.*, 2013), using ‘Cornell 49242’ as source of resistance. The *Co*-2 region (end of the Pv11) has been described previously (David *et al.*, 2009). In our study, the PWM2AS, mapped on Pv02, explained 7% of the phenotypic variation. Resistance genes and/or QTLs for many bean pathogens were found on Pv02, confirming the existence of R gene cluster on this chromosome (Hanai *et al.*, 2007; Oblessuc *et al.*, 2014; Campa *et al.*, 2014). Keller *et al.* (2015) reported a major QTL (ALS4.1^GS, UC^), localized on chromosome Pv04, that explained 75.3% of the ALS resistance.

The QTLs ALS11^AS^ and PWM11^AS^ presented the greatest effect on both diseases. These alleles derived from AND 277 are located within the same region on the Pv11, having the same marker linked (BAR5054) and close interval values (78.6–107.7 cM; 79.3–107.7 cM). However, for the ALS resistance, these alleles contributed for the reduction of the severity of the disease, while for PWM, they were associated with susceptibility. Thus, the AND 277 alleles conferred resistance to ALS, but contributed to the susceptibility to PWM. The presence of four resistance QTLs related to the response to ALS, resulting in a variable magnitude of phenotypic effects, indicated a complex pattern of inheritance for resistance to this disease in the AND 277 cultivar. Previous studies (Corrêa *et al.*, 2001; Faleiro *et al.*, 2004; Caixeta *et al.*, 2005) reported contrasting results supporting a monogenic pattern of inheritance for resistance to ALS. However, most of these reports evaluated the resistance in a qualitative fashion, forcing the classification of the genotypes into two distinct phenotypic classes (resistant or susceptible), a binomial distribution, instead of using the whole set of notes from the 0–9 scale.

Other QTL studies supported a quantitative nature of ALS resistance (Lopez *et al.*, 2003; Teixeira *et al.*, 2005; Mahuku *et al.*, 2011; Oblessuc *et al.*, 2012; Keller *et al.*, 2015). Mahuku *et al.* (2011), using a range of quantitative evaluations, found three genes for angular leaf spot resistance on the G5686 line and two on the G10909 line.

Our report provides the identification of new resistance loci for ALS and PWM resistance in common beans, revealing a quantitative pattern of inheritance to both diseases. The QTLs discovered in this study help to move bean resistance breeding toward a more efficient marker-assisted selection approach. The success of implementing a marker-assisted selection program depends on several factors such as a genetic map with molecular markers linked to genes controlling qualitative or quantitative traits of agronomic interest and a close association between markers and genes or QTL. Although the estimates presented here are for a particular breeding population, the common bean genome available on the Phytozome allows validating effective ALS and PWM resistance regions giving robustness to the estimates.

### Identification of putative resistance genes

BLAST searches (Tables 4 and 5) on the Phytozome revealed genes related to the immune response in plants such as glycosyl hydrolase, iron transporter, and receptor-like kinases (RLK).

**Table 4:** Gene predictions through BLAST search for powdery mildew-associated markers.

Marker	Pv(a)	E-value	Score	Chromosome position	Predicted gene	Distance (Kb)	Functional annotation	Homologs
BAR	2	6.1E-	416.1	31875852-31876085	Phvul.	4.4	U6 snRNA-associated Sm-like protein LSm7	Glyma.
								05G149300.1 (99%)
3703		115			002G171900.1			
								AT2G03870.1 (93.9%)
PVBR	2	8.00E-154	545.9	30942220-30942727	Phvul.	0	Glycosyl hydrolase family 35	Glyma.
								11G073100.1 (96.6%)
149					002G167200.1			
								AT4G36360.1 (86.9%)
BAR	11	2.2E-	457.5	41450864-41451120	Phvul.	3.2	PHD Finger Transcripton Factor	Glyma.
								13G272200.1 (94.0%)
5764		127			011G157300.1			
								AT4G22140.2 (87.5%)
BAR	11	1.8E-	417.9	45098859-45099093	Phvul.	7.6	D-mannose binding lectin // Protein tyrosine kinase // PAN-like domain	Glyma.
								08G125800.1 (63.4%)
5054		115			011G176300.1			
								AT1G65800.1 (47.5%)
BAR	11	1.1E-	338.5	49341224-49341414	Phvul.	0	Leucine-rich Repeat Receptor-like Protein Kinase	Glyma.
								12G235900.1 (85.6%)
5793		91			011G210400.1			
								AT2G21480.1 (73.2%)

a Chromossome

**Table 5:** Gene predictions through BLAST search for angular leaf spot-associated markers.

Marker	Pv(a)	*E-value*	Chromosome position	Predicted gene	Distance (Kb)	Functional annotation	Homologs
BAR	5	1.1E-111	37115819-37116054	Phvul.	0	RRM (RNA recognition motif)/	Glyma.13G334200.1 (90.2%)
4677				005G142200.1		nucleic acid binding	AT5G59950.1 (71.8%)
BAR	6	7.8E-127	26968242-26968500	Phvul.	6,5	Prenyltransferase and squalene oxidase repeat	Glyma.03G121300.2 (84.1%) AT1G78950.1 (75.6%)
6205				006G156700.1			
BAR	10	3.6E-131	12374562-12374825	Phvul.	31,5	Leucine-rich Repeat Receptor-like Protein Kinase	Glyma.01G125200.1 (88.6%) AT1G67510.1 (71.5%)
5437				010G064900.1			
BAR	10	1.00E-63	32153951-32154369	Phvul	25,8	C2 Domain-containing protein / Extended synaptotagmin-related	Glyma.07G082700.1 (91%) AT3G61050.2 (77%)
4576				.010G086200.1			
BAR	10	1.5 E ^-129^	10826301-10826557	Phvul.010G062000.1	4	No functional annotation	Glyma.01G128500.1 (85%) / AT2G38450.1 (69%)
5771							
BAR	10	1.8E-115	10960142-10960382	Phvul.	4,8	Iron transporter (Ferroportin1 (FPN1))	Glyma.01G128300.2 (86.3%) AT2G38460.1 (73.1%)
4354				010G062300.1			
BAR	10	2.8E-	11155767-11155985	Phvul.	56	F-Box protein, ATFBL3 / Leucine rich repeat proteins, some proteins contain F-box	Glyma.03G042600.1 (85%)
3550		68		010G062400.1			AT5G01720.1 (65.5%)
BAR	10	1.6E-	9994051-9994289	Phvul.	1,6	Leucine-rich Repeat Receptor-like Protein Kinase // Subfamily not named	Glyma.03G051100.1 (83.4%)
3032		59		010G060800.1			AT3G08870.1 (64.0%)
BAR	10	4.5E-104	22163551-22163791	Phvul.	9,2	Basic helix-loop-helix (BHLH) Family protein	Glyma.03G052300.1 (66,5%)
4606				010G073400.1			AT5G01310.1 (43.0%)
BAR	10	1.35E-	22288492-22288756	Phvul.	1,3	Phosphatidyl ethanolamine-binding protein	Glyma.01G123100.1 (89.9%)
6061		79		010G073500.1			AT5G01300.1 (78.6%)

a Chromossome

Limiting invasion by PWM in *A. thaliana* seem not to involve signaling molecules such as ethylene, jasmonic acid or salicylic acid, but requires a syntaxin, glycosyl hydrolase and ABC transporter (Consonni *et al.*, 2006). Here, the Phvul.002G167200 gene, coding a putative glycosyl hydrolase, was identified in the PWM2 QTL and contains the sequence of the PVBR149 marker (Figure 1 and Table 3). Its homolog in *Arabidopsis thaliana* (AT4G36360) was shown to respond to germinivirus (Ascencio-Ibañez *et al.*, 2008), indicating the importance of this gene/QTL to PWM resistance.

Cross talk between metal and biotic stress signaling is still not fully solved, but it is known that adequate intracellular concentrations of essential metal ions are required for pathogen virulence and plant defenses (Poschenrieder *et al.*, 2006).

RLKs are important pattern recognition receptors (PRRs) that play an important role in self- and non-self-recognition, including the perception of hormones (Shiu and Bleecker, 2001), PAMPs, and pathogen effectors. Several RLKs involved in plant immunity have been identified, such as *Xa*21 (Song *et al.*, 1998), Pto (Sessa *et al.*, 2000), Flagellin Sensing 2 (FLS2) (Chinchilla *et al.*, 2006) and BRASSINOSTEROID INSENSITIVE 1-ASSOCIATED KINASE 1 (BAK1) (Chinchilla *et al.*, 2007), among many others. This family of proteins has also been associated to ALS resistance (Keller *et al.*, 2015) in beans and for PWM resistance in wheat (Cao *et al.*, 2011). The RLKs identified in our study such as Phvul.010G064900 and Phvul.010G060800, associated to the ALS10 QTL, besides Phvul.011G176300 and Phvul.011G210400, positioned at the ALS11 and PWM11 QTLs constitute promising candidate genes for triggering the resistance response to ALS and PWM.

## Supplementary material

This online material is available for this article:

Table S1 - Diagrammatic scale notes used to evaluate the reaction of RILs to powdery mildew.

Figure S1 - Processing and analyzing of digital images of parent leaves.

Figure S2 - Distribution of powdery mildew (PWM) and angular leaf spot (ALS).
