# Supplementary material for: Linkage and mapping of quantitative trait loci associated with angular leaf spot and powdery mildew resistance in common beans
Source: Genet Mol Biol. 2017 Feb 20;40(1):109–22. doi: 10.1590/1678-4685-GMB-2015-0314 (PMC5409766; doi:10.1590/1678-4685-GMB-2015-0314)
Supplement: Supplementary file 1 [file 1415-4757-gmb-1678-4685-GMB-2015-0314-Suppl01.pdf]

Table S1. Diagrammatic scale notes used to evaluate the reaction of RILs to powdery mildew.

**Note**	**Severity**
1	Healthy leaves
2	0.5 a 10% of leaf area affected
3	11 – 25 % of leaf area affected
4	26 – 50% of leaf area affected
5	Lesions on stems and leaves
6	51 – 75% of leaf area affected
7	76 – 90% of leaf area affected
8	100% of yellow leaves
9	Dead leaves
