# Supplementary figures and images for: Linkage and mapping of quantitative trait loci associated with angular leaf spot and powdery mildew resistance in common beans
Source: Genet Mol Biol. 2017 Feb 20;40(1):109–22. doi: 10.1590/1678-4685-GMB-2015-0314 (PMC5409766; doi:10.1590/1678-4685-GMB-2015-0314)

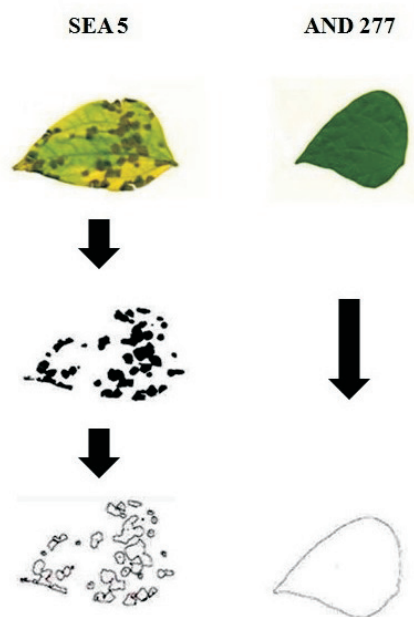

**Figure S1** - Processing and analyzing of digital images of parent leaves using ImageJ® software.

Supplement: Supplementary file 2 [file 1415-4757-gmb-1678-4685-GMB-2015-0314-Suppl02.pdf]
