# Supplementary material for: Linkage and mapping of quantitative trait loci associated with angular leaf spot and powdery mildew resistance in common beans
Source: Genet Mol Biol. 2017 Feb 20;40(1):109–22. doi: 10.1590/1678-4685-GMB-2015-0314 (PMC5409766; doi:10.1590/1678-4685-GMB-2015-0314)
Supplement: Supplementary file 3 [file 1415-4757-gmb-1678-4685-GMB-2015-0314-Suppl03.pdf]

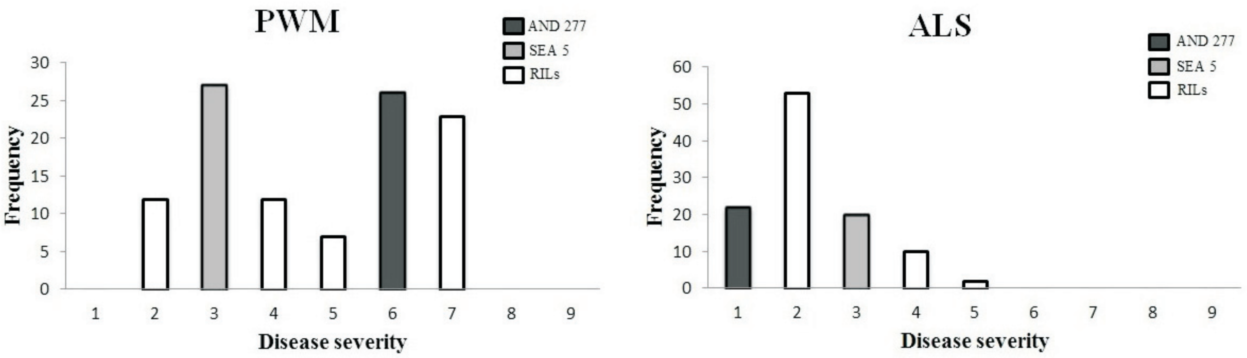


**Figure S2** - Distribution of powdery mildew (PWM) and angular leaf spot (ALS) severity scores (Least Square Means – LSMeans). Severity values of the parental lines are indicated by yellow (AND 277) and red (SEA 5) bars respectively.
